# Dual protective pathways: parallel mediating roles of Tangping tendency and positive self-esteem between gratitude and mental health among Chinese college students

**DOI:** 10.3389/fpsyg.2026.1853087

**Published:** 2026-06-16

**Authors:** Yangjie Wu, Junxing Pan

**Affiliations:** 1 Department of Education, Bozhou University, Bozhou, China; 2 Department of Students Affair, Huaibei Normal University, Huaibei, China

**Keywords:** mental health, parallel mediation, positive self-esteem, Tangping tendency, trait gratitude

## Abstract

This study investigated the relationship between trait gratitude and mental health among Chinese college students, with a specific focus on the potential mediating mechanisms of the Tangping (lying flat) tendency and positive self-esteem. A cross-sectional survey was administered to 828 undergraduate students in China, utilizing the Gratitude Questionnaire (GQ-6), the Tangping Questionnaire, the Positive Self-Esteem subscale of the Rosenberg Self-Esteem Scale, and the General Health Questionnaire (GHQ-12). Correlation analyses revealed significant associations among trait gratitude, the Tangping tendency, positive self-esteem, and mental health. Path analysis supported a parallel mediation model rather than a sequential one. Specifically, trait gratitude was associated with mental health not only directly, but also through two distinct and independent pathways: one involving lower Tangping tendencies, and another involving higher positive self-esteem. These findings suggest that gratitude is linked to college students’ mental well-being through dual, non-interfering mechanisms—lower passive coping behaviors alongside higher proactive self-evaluations. The results may inform targeted psychological interventions in higher education aimed at supporting resilience within competitive academic environments.

## 1 Introduction

In recent years, higher education systems globally have witnessed a notable shift in students’ achievement motivation and coping strategies. During the transition to early adulthood, college students face multiple compounding stressors—ranging from academic demands to career uncertainty—that render them highly susceptible to psychological distress (Deng et al., 2022). Driven by societal transitions, public health crises, and escalating hyper-competition (often conceptualized as “involution” or neijuan), the psychological burden on youth has become increasingly complex (Shu et al., 2021; Wang et al., 2020). In response to these relentless pressures, a pervasive socio-psychological phenomenon of behavioral withdrawal and anti-achievement orientation has emerged among Generation Z. This global zeitgeist manifests diversely as “quiet quitting” in Western contexts (Formica and Sfodera, 2022) and the “Tangping” (lying flat) subculture in China (Teng, 2022; Lu et al., 2024). While traditional dual-factor models of mental health primarily focus on mitigating psychopathology and promoting subjective well-being as a vital resilience buffer (Lamers et al., 2015; Wang and Zhang, 2011), they often overlook these emerging defensive postures. Consequently, from a positive psychology perspective, understanding how core protective traits like gratitude may be associated with better mental health—not by coercing students back into toxic competition, but by buffering them within this modern context of voluntary psychological detachment—holds profound theoretical value.

Among various positive psychological resources, gratitude has been consistently identified as a core protective factor for promoting and maintaining mental health (Wood et al., 2010), particularly within educational settings (Bono et al., 2022). Gratitude is conceptualized as a stable trait reflecting one’s tendency to recognize and respond with positive emotions to the benevolent actions of others or external circumstances (McCullough et al., 2002). According to the broaden-and-build theory of positive emotions (Fredrickson, 2001, 2004a,b), gratitude not only “broadens” an individual’s momentary thought-action repertoires—enabling more flexible cognitive processing under stress—but also helps “build” enduring psychological and social capital. Recent meta-analyses and large-sample studies consistently confirm that trait gratitude consistently predicts psychological well-being, effectively buffering against psychopathological symptoms (Jans-Beken et al., 2020; Portocarrero et al., 2020). Based on this, we propose:

Hypothesis 1 (*H1*): Gratitude positively predicts the mental health of college students.

To further elucidate the underlying mechanisms through which gratitude shows beneficial associations, this study introduces “Tangping” (colloquially termed “lying flat”)—a pervasive contemporary socio-psychological phenomenon among Chinese youth (Teng, 2022). Contextualized within a hyper-competitive educational climate, we specifically conceptualize and operationalize Tangping as a maladaptive coping posture characterized by deliberate amotivation and behavioral withdrawal (Lu et al., 2024). To situate this construct within the broader international psychological discourse, Tangping shares conceptual cognates with established Western frameworks, such as academic burnout (Schaufeli et al., 2002) and amotivation within Self-Determination Theory (Legault et al., 2006; Howard et al., 2021), and parallels the recent global trend of “quiet quitting” (Formica and Sfodera, 2022).

To establish the discriminant validity of Tangping as a distinct psychological construct, it is essential to delineate its conceptual boundaries from six related but distinct Western constructs. First, unlike academic burnout, which is primarily driven by involuntary emotional exhaustion resulting from prolonged academic demands (Madigan and Curran, 2021; Schaufeli et al., 2002), Tangping represents a voluntary, value-driven withdrawal—individuals consciously choose to lower their achievement aspirations rather than being depleted into incapacity. Second, whereas learned helplessness arises from a perceived lack of control over outcomes (Seligman, 1972), Tangping entails preserved agency: individuals retain the perceived capacity to compete but deliberately opt out of what they view as a system of diminishing returns. Third, amotivation within Self-Determination Theory reflects an absence of motivation (Legault et al., 2006; Howard et al., 2021), whereas Tangping is itself a motivated choice—a strategic recalibration of values away from hyper-competitive norms rather than a deficit in motivation. Fourth, general academic disengagement denotes a passive withdrawal from scholastic tasks and goals, while Tangping is specifically targeted at the competitive, involutionary (neijuan) dimension of academic life. Fifth, psychological detachment from work or study serves a healthy recovery function that restores resources (Sonnentag and Fritz, 2015), whereas Tangping reflects a more sustained value reorientation that, while potentially offering short-term psychological relief, carries uncertain long-term consequences for development and goal attainment. Sixth, the recent global phenomenon of “quiet quitting”—doing the minimum required without going above and beyond (Formica and Sfodera, 2022)—is primarily framed as a workplace behavior, whereas Tangping extends beyond specific role performance into a broader value orientation and life philosophy among Chinese youth, one that is deeply intertwined with reactions to neijuan and the perceived futility of hyper-competition. In summary, Tangping is characterized by three core features that differentiate it from these existing constructs: (a) it is a voluntary, value-driven choice rather than an involuntary deficit; (b) it is socio-culturally embedded within China’s hyper-competitive academic environment (neijuan), reflecting a collective disillusionment with achievement norms that are reinforced by familial expectations and social comparison; and (c) it reflects a shift in life values and meaning systems, not merely a behavioral reduction in effort. With this conceptual clarification in place, we now turn to the theoretical mechanisms that may explain how gratitude relates to Tangping, self-esteem, and mental health.

Having established the conceptual boundaries of Tangping, we now examine the socio-cultural forces that give rise to it in the Chinese higher education context. Three interconnected forces are particularly salient. First, the legacy of the Gaokao—China’s high-stakes college entrance examination—instills an enduring achievement-oriented mindset, wherein academic performance becomes inextricably linked to personal worth and family honor. Students who have spent their formative years in an intensely competitive examination culture may experience acute dissonance upon entering university, where the singular metric of test scores gives way to diffuse and often opaque criteria for success. Second, the phenomenon of neijuan (involution)—a state of diminishing returns from intensified competition—places students under intensifying pressure, as they invest escalating effort into academic and extracurricular activities with proportionally declining rewards (Lu et al., 2024). This structural dynamic fosters a sense of futility that directly fuels the Tangping posture: if exerting maximum effort yields no meaningful advantage, disengagement becomes a rational and psychologically self-protective response. Third, these pressures are amplified by Confucian cultural values, particularly filial piety and the pursuit of family honor through educational achievement. When such expectations confront a constrained social mobility landscape—marked by rising youth unemployment, escalating housing costs, and a perceived ceiling on upward mobility—Tangping emerges not merely as individual withdrawal, but as a collective, culturally embedded renegotiation of what constitutes a meaningful and viable life path. These forces distinguish Tangping from superficially similar Western phenomena and underscore the importance of examining culturally specific protective factors, such as gratitude, that may buffer Chinese students against these unique pressures. These cultural dynamics provide the backdrop against which psychological resources like gratitude may serve protective functions—a possibility we explore through the theoretical lenses below.

Drawing upon Conservation of Resources (COR) theory (Hobfoll, 1989) and Self-Determination Theory (SDT; Ryan and Deci, 2017), we propose a cohesive mechanism: trait gratitude functions as a robust psychological reservoir that promotes the use of adaptive coping styles (Wood et al., 2007), which not only buffers against the perceived depletion of intense competition but also facilitates the satisfaction of basic psychological needs for autonomy and competence. Consequently, gratitude endows individuals with the resilient resources necessary to proactively engage with academic adversity, thereby neutralizing the tendency to adopt the defensive, disengaged posture of Tangping. Thus, we propose:

Hypothesis 2 (*H2*): Tangping (lying flat) mediates the relationship between gratitude and mental health.

In addition to its association with behavioral coping strategies, gratitude is also associated with mental health through its relation to the individual’s self-cognitive system, wherein positive self-esteem plays a pivotal role. Positive self-esteem refers to an individual’s proactive, affirmative evaluation of their own worth and competence. Extensive longitudinal studies have established self-esteem as a core cognitive indicator for maintaining optimal mental health (Orth and Robins, 2013). In the present study, we focus specifically on its positive dimension—proactive self-affirmation—which may be particularly relevant to the resource-building function of gratitude. Guided by the broaden-and-build theory and its specific application to gratitude (Fredrickson, 2001, 2004a, 2004b), we propose that such sustained experiences help individuals construct positive self-schemas and proactively expand their sense of self-worth. Specifically, meta-analyses across diverse cultural contexts have consistently demonstrated a robust correlation between dispositional gratitude and indicators of enhanced psychological well-being, including positive self-evaluations (Portocarrero et al., 2020).”Therefore, aligning with the conceptual focus on proactive resource building, we propose:

Hypothesis 3 (*H3*): Positive self-esteem mediates the relationship between gratitude and mental health.

The proposed mediation model positions gratitude as the upstream predictor based on several converging theoretical rationales. First, within the broaden-and-build framework (Fredrickson, 2001), gratitude—as a prototypical positive emotional trait—broadens attentional and cognitive repertoires, which in turn enables the accumulation of durable personal resources such as positive self-esteem, rather than the reverse. Second, Conservation of Resources theory (Hobfoll, 1989) conceptualizes gratitude as a personal resource that precedes and buffers against resource loss; individuals with lower gratitude are thus theorized to be more susceptible to defensive withdrawal (Tangping), rather than adopting withdrawal because they lack gratitude. Third, from an implicit theories perspective (Dweck, 2006), gratitude is considered a malleable character strength that can be intentionally cultivated, positioning it as an active antecedent that shapes downstream psychological outcomes rather than a passive reflection of existing well-being. Fourth, character strength theory (Peterson and Seligman, 2004) classifies gratitude as a “transcendence” strength—an upstream virtue that orients individuals toward meaning and connection, thereby facilitating positive self-evaluation and mental health. Collectively, these frameworks provide converging support for the theoretical plausibility of the gratitude → mediators → mental health pathway examined in the present study. Nevertheless, given the cross-sectional design, alternative temporal orderings cannot be definitively ruled out; the present model represents the most theoretically grounded configuration pending longitudinal verification.

A theoretical link may also exist between the Tangping tendency and positive self-esteem. According to the framework of Social Cognitive Theory (Bandura, 1986), active engagement in behavioral practices and goal pursuit primarily cultivates self-efficacy, which subsequently contributes to the development of a positive self-concept. Chronically adopting the “lying flat” strategy entails a voluntary abandonment of goal pursuit and competence validation. “Regarding the logical sequence of this potential pathway, the ‘loss spiral’ concept within Conservation of Resources (COR) theory (Hobfoll, 1989) suggests that behavioral coping might precipitate changes in cognitive evaluation: initial resources (or lack thereof) shape defensive behavioral coping postures like Tangping (Lu et al., 2024). However, we hypothesize that these withdrawal responses may subsequently deprive individuals of the experiential feedback necessary to maintain their internal positive self-esteem.” Based on this theoretical deduction, we formulate a sequential mediation hypothesis to be rigorously tested against our empirical data:

Hypothesis 4 (*H4*): The Tangping tendency and positive self-esteem play a sequential mediating role in the relationship between gratitude and mental health.

This study offers specific, multi-level contributions to the existing literature. Theoretically, by operationalizing a pervasive sociocultural phenomenon (Tangping) as a testable psychological construct, this study bridges sociological discourse with empirical psychological science. By rigorously testing these complex mediational pathways, this research clarifies the specific structural mechanisms—investigating whether gratitude is associated with mental health through a sequential spillover effect (where behavioral withdrawal inevitably erodes self-worth) or through robust parallel reservoirs. Practically, uncovering the precise nature of these structural relationships (sequential vs. parallel) is critical for higher education institutions to design targeted, evidence-based psychological interventions that can effectively build college students’ resilience amidst a hyper-competitive academic climate.

## 2 Materials and methods

### 2.1 Participants and procedure

A convenience sampling method was used to recruit undergraduate students from two universities in Anhui Province, China. Data collection took place between June 2 and June 21, 2024. Questionnaires were distributed by instructors in classroom settings during lecture hours. Prior to the survey, participants were presented with a clear statement detailing the research objectives, the voluntary nature of participation, and their right to withdraw at any time. By proceeding to complete and submit the questionnaire, participants provided their informed consent. All data were collected and processed anonymously to ensure participant confidentiality and data integrity.

A total of 840 questionnaires were collected. Following the exclusion of 12 invalid responses characterized by excessively short completion times or patterned answering (e.g., straight-lining), 828 valid responses were retained for the final analysis, yielding an effective response rate of 98.6%. The final sample consisted of 666 females (80.4%) and 162 males (19.6%), with a mean age of 20.24 years (*SD* = 1.40). In terms of academic grade, there were 317 freshmen (38.3%), 260 sophomores (31.4%), 207 juniors (25.0%), and 44 seniors (5.3%). The distribution across academic disciplines included humanities and social sciences (*n* = 650, 78.5%), STEM fields (*n* = 122, 14.7%), and other areas (*n* = 56, 6.8%). Regarding the participants’ backgrounds, 560 students (67.6%) were from rural areas, while 268 (32.4%) were from urban areas. Given the convenience sampling method, the sample was predominantly composed of female students from humanities and social science disciplines; the potential implications of this demographic distribution on the study’s external validity are addressed in the limitations section.

### 2.2 Measures

Prior to hypothesis testing, Confirmatory Factor Analyses (CFA) were conducted for all primary measurement scales to evaluate their construct validity. For the Gratitude Questionnaire (GQ-6), the initial single-factor model showed poor fit due to method variance introduced by the two reverse-worded items. Following modification indices, one error covariance was freely estimated between the two reverse-scored items (items 3R and 6R; see Wei et al., 2011) to account for this well-documented wording effect. The modified model demonstrated acceptable fit: χ^2^(8) = 61.43, χ^2^/df = 7.68, CFI = 0.978, TLI = 0.959, RMSEA = 0.090 [90% CI: 0.070, 0.111], SRMR = 0.027. For the Positive Self-Esteem (PSE) subscale, the single-factor model with six positively worded items demonstrated acceptable fit without any post-hoc modifications: χ^2^(9) = 160.74, χ^2^/df = 17.86, CFI = 0.942, TLI = 0.904, RMSEA = 0.143 [90% CI: 0.124, 0.162], SRMR = 0.038. For the Tangping scale, a two-factor model corresponding to its two sub-dimensions (“Cognitive Awakening” and “Behavioral Escapism”) was specified. Based on modification indices, three error covariances were allowed between items within the same subscale: items 11 and 9 (both reflecting reduced material aspirations), items 3 and 5 (both reflecting preference for a simpler lifestyle), and items 7 and 6 (both reflecting academic withdrawal). The modified model demonstrated acceptable fit: χ^2^/df = 7.45, CFI = 0.914, TLI = 0.886, RMSEA = 0.088, SRMR = 0.062. For the GHQ-12, a two-factor model (positive and negative health dimensions) was specified, resulting in good fit: χ^2^/df = 5.05, CFI = 0.963, TLI = 0.952, RMSEA = 0.070, SRMR = 0.053. Following established guidelines, acceptable model fit was determined by CFI and TLI values ≥ 0.90, SRMR ≤ 0.08, and RMSEA ≤ 0.08 (Hu and Bentler, 1999).

#### 2.2.1 Gratitude

Gratitude was assessed using the 6-item Gratitude Questionnaire (GQ-6; McCullough et al., 2002), validated in Chinese by Wei et al. (2011). Participants rated items on a 7-point Likert scale (1 = strongly disagree, 7 = strongly agree). After reversing negatively worded items, a higher total score indicated a stronger dispositional gratitude. The modified CFA results for the unidimensional structure indicated an excellent model fit: χ2/df = 7.68, CFI = 0.978, TLI = 0.959, SRMR = 0.027, and RMSEA = 0.090. Although the RMSEA value slightly exceeded the conventional 0.08 threshold, this may partly reflect the model’s small degrees of freedom (Kenny et al., 2015); the other fit indices (CFI = 0.978, SRMR = 0.027) indicated excellent fit. The scale demonstrated acceptable internal consistency (Cronbach’s *α* = 0.70).

#### 2.2.2 Tangping (lying flat) tendency

Tangping tendency was measured using the 12-item Tangping Questionnaire, developed by Li et al. (2022). Responses were recorded on a 5-point Likert scale ranging from 1 (strongly disagree) to 5 (strongly agree). To ensure cross-cultural construct validity and theoretical rigor, it is imperative to note that while this instrument was originally developed within a Chinese socio-cultural context, its core dimensions conceptually map onto established Western psychological constructs. Specifically, its two sub-dimensions—"Cognitive Awakening” (a critical reappraisal of relentless competition) and “Behavioral Escapism” (disengagement from conventional achievement metrics)—closely parallel the concepts of “psychological detachment” (Sonnentag and Fritz, 2015) and “amotivation” within Self-Determination Theory (Ryan and Deci, 2017). By capturing this deliberate, defensive disengagement, the scale provides a psychometrically sound operationalization of the global “quiet quitting” phenomenon. Although the scale encompasses these two sub-dimensions, the composite total score was utilized in the current mediation analysis to represent the overarching behavioral and motivational withdrawal tendency, with a higher average score reflecting a more severe disengaged posture. The modified measurement model in the current sample demonstrated an acceptable structural fit: χ2/df = 7.45, CFI = 0.914, TLI = 0.886, SRMR = 0.062, and RMSEA = 0.088. The RMSEA value slightly exceeded the conventional 0.08 cutoff but should be interpreted with caution given the model’s degrees of freedom (Kenny et al., 2015); the CFI (0.914) and SRMR (0.062) suggested acceptable overall fit. The overall internal consistency was excellent (Cronbach’s *α* = 0.87).

#### 2.2.3 Positive self-esteem

Positive self-esteem was evaluated using the Rosenberg Self-Esteem Scale (RSES; Rosenberg, 1965). While traditionally conceptualized as a unidimensional construct, extensive cross-cultural psychometric evaluations have demonstrated that the RSES frequently bifurcates into positive and negative dimensions within Chinese populations, largely driven by the method artifacts associated with reverse-scored items (Tian, 2006). Given the well-documented concerns that reverse-worded items in the RSES may introduce construct-irrelevant method variance in Chinese samples (Tian, 2006), only the positively worded items were retained for the present analysis, consistent with psychometric recommendations for this population. To rigorously ensure structural validity and eliminate methodological confounding, we exclusively extracted the 6-item Positive Self-Esteem (PSE) subscale (items 1, 2, 4, 6, 7, and positively-scored item 8) for the current path analysis, reflecting an individual’s proactive self-affirmation. The Confirmatory Factor Analysis (CFA) for this pure positive dimension demonstrated excellent incremental and absolute fit indices: χ2/df = 17.86, CFI = 0.942, TLI = 0.904, and SRMR = 0.038. The RMSEA value (0.143) was elevated, which may partly reflect the known tendency of RMSEA to be overstated in models with small degrees of freedom (df = 9; Kenny et al., 2015). In such cases, SRMR and CFI are considered more reliable indicators of model fit. The SRMR (0.038) and CFI (0.942) values suggested acceptable fit; nevertheless, the elevated RMSEA should be acknowledged as a potential measurement limitation. The internal consistency for the positive subscale was excellent (Cronbach’s *α* = 0.89). Accordingly, all references to “self-esteem” in the present study pertain specifically to the positive dimension of self-evaluation (i.e., proactive self-affirmation) rather than global self-esteem; findings should not be generalized to the full Rosenberg Self-Esteem Scale.

#### 2.2.4 Mental health

Mental health was assessed using the 12-item General Health Questionnaire (GHQ-12), revised for Chinese populations by Li and Li (2015). The questionnaire includes positive and negative health dimensions. Participants rated items on a 4-point scale. To facilitate intuitive interpretation and align with the positive psychology framework of this study, negative items were reverse-scored, and positive items were scored straightforwardly. Thus, contrary to the traditional GHQ-12 scoring, higher overall scores in this study were intentionally transformed to denote better mental health status. The modified two-factor structure demonstrated an excellent fit: χ^2^/*df* = 5.05, CFI = 0.963, TLI = 0.952, SRMR = 0.053, and RMSEA = 0.070. The overall Cronbach’s α for the GHQ-12 was 0.79.

### 2.3 Statistical analysis

*A priori* power analysis using G^*^Power 3.1 indicated that a sample size of 828 provides sufficient statistical power (> 0.95) to detect small-to-medium indirect effects in mediation models. Data screening, descriptive statistics, and Pearson correlation coefficients were conducted using SPSS 25.0. Data normality was confirmed (skewness and kurtosis within acceptable ranges). Multicollinearity was checked, with Variance Inflation Factor (VIF) values well below the threshold of 5.

Although procedural remedies were applied to minimize common method variance (CMV), Harman’s single-factor test was still conducted as a preliminary statistical check. The sequential mediation model was tested using Model 6 of the PROCESS macro for SPSS (Hayes, 2022). We explicitly tested the specified sequential path: Gratitude → Tangping → Positive self-esteem → Mental health. To ensure the robustness of the indirect effects, bootstrapping with 5,000 resamples was performed to calculate 95% confidence intervals (CIs).

Finally, preliminary analyses (independent samples t-tests, one-way ANOVAs, and Pearson correlations) were conducted to examine the potential confounding effects of demographic variables (gender, academic year, residential background, discipline, and age). Results indicated no significant differences or correlations across any demographic categories concerning the dependent variable (mental health, all *p*s > 0.05). However, further analyses revealed that specific demographic variables significantly influenced the mediator variables. Specifically, gender exhibited a significant effect on both the Tangping tendency (*p* < 0.001) and positive self-esteem (*p* = 0.004), while academic discipline significantly affected positive self-esteem (*p* = 0.025). Other demographic variables (academic year, residential background, and age) showed no significant effects. Consequently, to rigorously control for these potential confounding effects and ensure the robustness of the results, gender and academic discipline were retained as covariates in the subsequent mediation analyses, whereas the non-significant demographic variables were excluded to maintain model parsimony.

## 3 Results

### 3.1 Preliminary analyses

Prior to testing the primary hypotheses, preliminary analyses were conducted to ensure data quality and satisfy the statistical assumptions required for the mediation model. First, Harman’s single-factor test was performed to check for common method variance (CMV). The unrotated exploratory factor analysis extracted 24 factors with eigenvalues greater than 1. The first principal factor accounted for only 20.98% of the total variance, which is well below the established threshold of 40%, indicating that CMV is not a serious concern in this study. However, given the inherent limitations of statistical post-hoc remedies, procedural controls (e.g., anonymous responding, reversed items) implemented during data collection serve as the primary defense against CMV.

Second, the normality of the data was assessed. As shown in Table 1, the absolute values of skewness for all primary variables ranged from 0.015 to 0.591, and the absolute values of kurtosis ranged from 0.136 to 0.916. Given the large sample size (*N* = 828) and the use of the bootstrapping method, which is robust against non-normality, these values (strictly skewness < 3 and kurtosis < 10) confirm that the data adequately approximate a normal distribution. Multicollinearity was also examined; the Variance Inflation Factor (VIF) values for all predictors were well below the stringent threshold of 5 (ranging from 1.06 to 1.20), indicating no severe multicollinearity issues.

**Table 1:** Descriptive statistics and correlations among study variables (*N* = 828).

Variables	*M* ± *SD*	1	2	3	4
1. Gratitude	5.13 ± 0.89	1			
2. Tangping tendency	3.01 ± 0.66	−0.16**	1		
3. Positive self-esteem	3.07 ± 0.46	0.29**	−0.01	1	
4. Mental health	2.74 ± 0.43	0.32**	−0.13**	0.48**	1

M = Mean; SD = Standard Deviation. **p < 0.01.

Finally, preliminary analyses (independent samples t-tests, one-way ANOVAs, and Pearson correlations) were conducted to examine the potential confounding effects of demographic variables (gender, academic year, residential background, discipline, and age). Results indicated no significant differences or correlations across any demographic categories concerning the dependent variable (mental health, all *p*s > 0.05). However, further analyses revealed that specific demographic variables significantly influenced the mediator variables. Specifically, gender exhibited a significant effect on both the Tangping tendency (*p* < 0.001) and positive self-esteem (*p* = 0.004), while academic discipline significantly affected positive self-esteem (*p* = 0.025). Other demographic variables (academic year, residential background, and age) showed no significant effects. Consequently, to rigorously control for these potential confounding effects and ensure the robustness of the results, gender and academic discipline were dummy coded (e.g., male = 0, female = 1; discipline with k-1 coding) and retained as covariates in the subsequent mediation analyses, whereas the non-significant demographic variables were excluded to maintain model parsimony.

### 3.2 Descriptive statistics and correlational analysis

The descriptive statistics and Pearson correlation coefficients for all primary variables are summarized in Table 1. As hypothesized, gratitude was significantly and negatively correlated with the Tangping tendency (*r* = −0.16, *p* < 0.01) and significantly and positively correlated with both positive self-esteem (*r* = 0.29, *p* < 0.01) and mental health (*r* = 0.32, *p* < 0.01). The Tangping tendency exhibited a significant negative correlation with mental health (*r* = −0.13, *p* < 0.01), while positive self-esteem demonstrated a robust positive correlation with mental health (*r* = 0.48, *p* < 0.01). The bivariate correlation between the Tangping tendency and positive self-esteem was non-significant (*r* = −0.01, *p* = 0.704). This empirical “decoupling” between behavioral withdrawal and internal self-evaluation provides strong preliminary evidence against a sequential mediation mechanism. Instead, it suggests that Tangping and positive self-esteem may function as independent, parallel pathways through which gratitude influences mental health, thereby providing a firm statistical foundation for the subsequent parallel mediation analysis.

### 3.3 Testing the sequential mediation model

**Figure 1:**
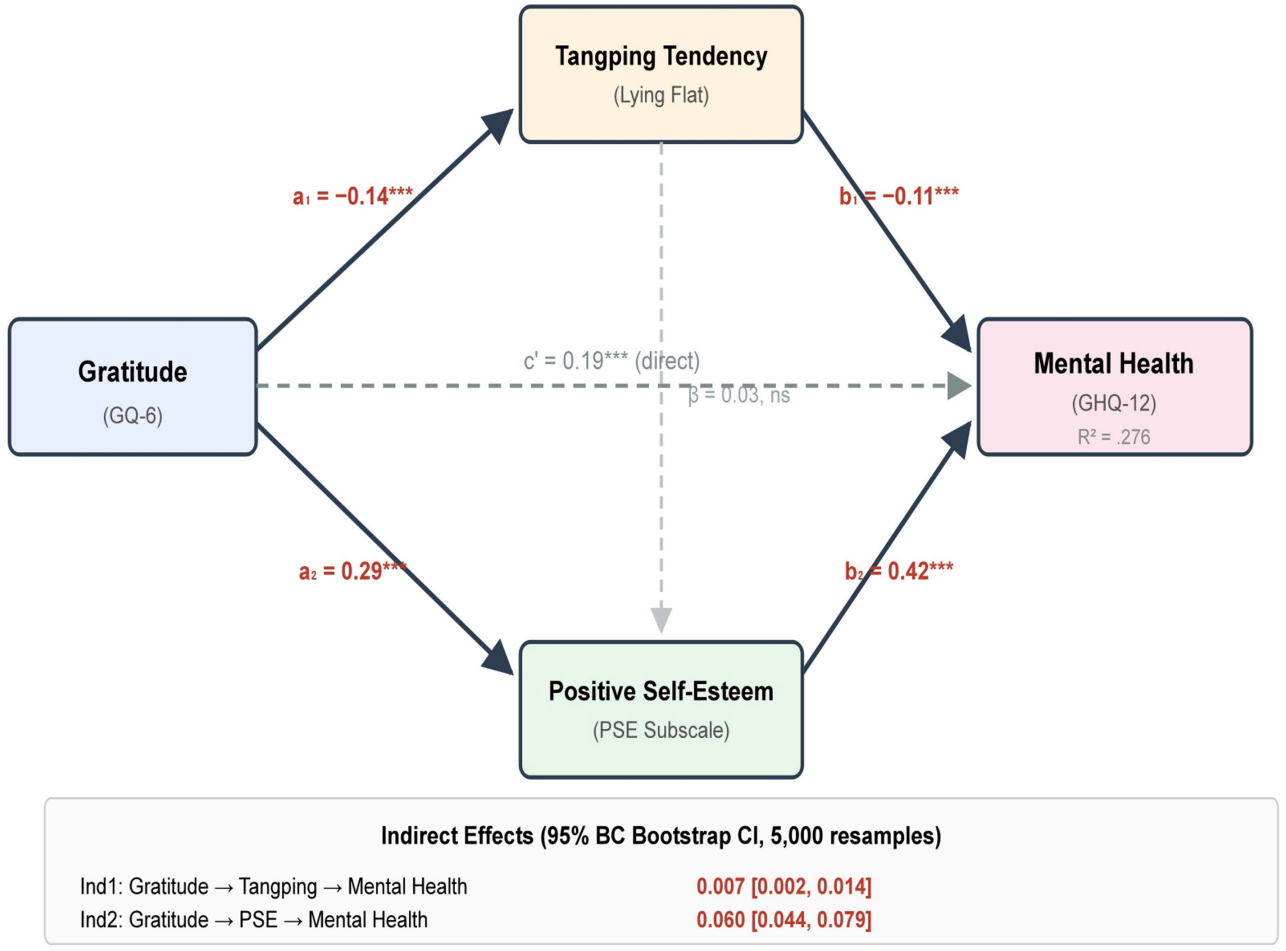
The parallel mediation model depicting the hypothesized pathways: gratitude is associated with mental health through two independent mediating mechanisms—the Tangping tendency and positive self-esteem.

The sequential mediation model was tested using Model 6 of the PROCESS macro. After controlling for gender and academic discipline, the results revealed a nuanced mechanism of how gratitude influences mental health. Firstly, gratitude significantly and negatively predicted the Tangping tendency (*β* = −0.14, *p* < 0.001) and positively predicted positive self-esteem (*β* = 0.29, *p* < 0.001). In the final model predicting mental health, gratitude (*β* = 0.19, *p* < 0.001), Tangping tendency (*β* = −0.11, *p* < 0.001), and positive self-esteem (*β* = 0.42, *p* < 0.001) all emerged as significant predictors, collectively explaining 27.6% of the variance in mental health. However, the hypothesized sequential path from Tangping to positive self-esteem was not statistically significant (*B* = 0.02, *p* = 0.341), indicating that the behavioral withdrawal of “lying flat” did not necessarily erode students’ positive self-evaluations. Consequently, the sequential mediation effect (Ind3: Gratitude→ Tangping→ Positive Self-Esteem→ Mental Health) was not supported (Effect = −0.0009, 95% CI [−0.0036, 0.0019]). Instead, the results demonstrated a parallel mediation mechanism. Two distinct indirect pathways were identified: (1) the behavioral path, where gratitude promoted mental health by reducing the Tangping tendency (Ind1: Effect = 0.007, 95% CI [0.0018, 0.0136]); and (2) the cognitive path, where gratitude enhanced mental health through its linkage with positive self-esteem (Ind2: Effect = 0.060, 95% CI [0.0437, 0.0788]). Among these, the cognitive path (Positive Self-Esteem) exhibited a substantially stronger mediating effect, see Table 2 and Figure 1.

**Table 2:** Regression results for the sequential mediation model controlling for covariates.

Predictors	*β*	*SE*	*t*	*p*	*R* ^2^	*F*
Model 1: Predicting Tangping tendency					0.046	9.84***
Gratitude	−0.14	0.03	−4.05	<0.001		
Model 2: Predicting positive self-esteem					0.089	16.12***
Gratitude	0.29	0.02	8.62	<0.001		
Tangping tendency	0.03	0.02	0.95	0.341		
Model 3: Predicting mental health					0.276	52.15***
Gratitude	0.19	0.02	5.96	<0.001		
Tangping tendency	−0.11	0.02	−3.45	<0.001		
Positive self-esteem	0.42	0.03	13.61	<0.001		

*N* = 828. *β* represents standardized regression coefficients. SE represents the standard error of the unstandardized coefficients. Gender and academic discipline were controlled as covariates in all models. ***p < 0.001.

Furthermore, bootstrapping procedures with 5,000 resamples were utilized to test the significance of the specific indirect effects (see Table 3). The total indirect effect of gratitude on mental health was significant (Effect = 0.066, 95% CI [0.050, 0.086]). Specifically, the mediation analysis revealed the following distinct pathways:

**Table 3:** Indirect effects of gratitude on mental health.

Pathways	Effect	Boot SE	Boot LLCI	Boot ULCI
Total indirect effect	0.066	0.009	0.05	0.086
Ind1: Gratitude → Tangping → Mental health	0.007	0.003	0.002	0.014
Ind2: Gratitude → Positive self-esteem → Mental health	0.06	0.009	0.044	0.079
Ind3: Gratitude → Tangping → Positive self-esteem → Mental health	–0.001	0.001	–0.004	0.002

Boot SE = bootstrapped standard error; LLCI = lower limit of 95% confidence interval; ULCI = upper limit of 95% confidence interval. Unstandardized effects are reported.

Ind1 (Gratitude → Tangping → Mental Health): The independent mediating effect of the Tangping tendency was significant, as the 95% CI did not include zero (Effect = 0.007, 95% CI [0.002, 0.014]). This indicates that gratitude is significantly associated with lower Tangping tendencies, which are in turn independently associated with mental health. Ind2 (Gratitude → Positive Self-Esteem → Mental Health): Positive self-esteem significantly and independently mediated the relationship between gratitude and mental health (Effect = 0.060, 95% CI [0.044, 0.079]), supporting Hypothesis 3. This pathway accounted for the largest proportion of the total indirect effect. Ind3 (Gratitude → Tangping → Positive Self-Esteem → Mental Health): The sequential mediating pathway was not statistically significant, as the 95% CI included zero (Effect = −0.001, 95% CI [−0.004, 0.002]). This result aligns with the non-significant path from Tangping to positive self-esteem observed in Model 2. Thus, Hypothesis 4, which proposed a sequential mediation mechanism, was not supported. Synthesizing these findings, the sequential mediation hypothesis is rejected in favor of a parallel mediation model. The results indicate two distinct and independent psychological pathways: a behavioral path (lower Tangping tendencies) and a cognitive path (higher positive self-esteem), both of which are convergently associated with the mental health of college students.

## 4 Discussion

This study constructed and tested a sequential mediation model to comprehensively explore the underlying mechanisms by which gratitude, the Tangping tendency, and positive self-esteem are collectively associated with mental health among college students. The findings elucidate the multidimensional factors affecting mental health and clarify the complex pathways through which gratitude operates, providing robust empirical evidence for higher education interventions.

### 4.1 The relationship between gratitude and mental health

Our findings revealed that gratitude significantly and positively predicts mental health, which strongly supports Hypothesis 1. The standardized total effect of gratitude on mental health (*β* = 0.33) indicates a moderate effect size, which is highly consistent with recent meta-analytic findings on gratitude and well-being (Portocarrero et al., 2020).

The broaden-and-build theory of positive emotions provides a robust theoretical framework for these results (Fredrickson, 2001, 2004a). As a core positive emotion, gratitude broadens an individual’s momentary thought-action repertoires and builds enduring personal resources, which directly promote mental well-being (Fredrickson, 2004b). Furthermore, validating this protective role within a Chinese college student sample contributes to the existing literature by providing further empirical support for the applicability of Western positive psychology theories within East Asian higher education contexts characterized by high competition and academic “involution” (neijuan).

### 4.2 The independent mediating role of positive self-esteem

Prior to interpreting the mediating mechanism, the present model specifically focalizes on the positive dimension of self-esteem (i.e., proactive self-affirmation). Methodologically, extracting this pure sub-dimension successfully circumvents the well-documented construct-irrelevant method artifacts associated with reverse-worded items in the Rosenberg Self-Esteem Scale among Chinese populations (e.g., Tian, 2006). Theoretically, this specification aligns seamlessly with the broaden-and-build framework. Gratitude, as a resource-accumulating trait, inherently involves a proactive cognitive reframing process—focusing on building positive psychological resources rather than merely dampening negative distress (Lambert et al., 2012). When this cognitive reframing is directed inward toward the self, gratitude is associated with more positive self-schemas and greater self-worth, consistent with meta-analytic evidence on the gratitude–well-being relationship (Portocarrero et al., 2020). Consequently, employing positive self-esteem serves as a precise, conceptually congruent cognitive indicator of the psychological dividends yielded by trait gratitude. Building upon this conceptualization, our data demonstrated that positive self-esteem significantly and independently mediates the relationship between gratitude and mental health, strictly verifying Hypothesis 3. This resonates with the broaden-and-build theory, suggesting that gratitude fosters the robust development of positive self-esteem as a vital psychological reservoir (Fredrickson, 2004a, 2004b). Although prior research on these mediating pathways has primarily employed global measures of self-esteem (Bajaj et al., 2016; Kong et al., 2015; Lin, 2015), the present findings suggest that even the positive dimension alone serves as a robust cognitive mediator between positive traits and mental health outcomes. Moreover, this specific cognitive pathway accounted for the vast majority of the total indirect effect, underscoring its pivotal role in the protective mechanism. This finding aligns with the broader vulnerability model of psychopathology, which has established that low self-esteem—conceptualized globally—increases risk for psychological distress (Orth and Robins, 2013; Zeigler-Hill, 2011). The present data extend this model by demonstrating that even the positive dimension specifically may serve as a cognitive buffer against psychological distress. Notably, the direct effect of gratitude on mental health remained significant (*β* = 0.19, *p* < 0.001) even after controlling for the mediators, hinting at the existence of other parallel protective mechanisms (e.g., enhanced perceived social support) that warrant future empirical investigation.

### 4.3 The sequential mediating roles of the Tangping tendency and positive self-esteem

Contrary to Hypothesis 4, the results did not support a sequential mediation involving the Tangping tendency and positive self-esteem. Specifically, while gratitude was associated with lower Tangping tendencies and higher positive self-esteem independently, the structural path from Tangping to positive self-esteem was statistically non-significant (*β* = 0.03, *p* = 0.341). This indicates that among Chinese college students, behavioral withdrawal (Tangping) does not necessarily entail a concomitant erosion of self-worth. This empirical “decoupling” of behavior and self-evaluation provides a highly nuanced extension of Social Cognitive Theory within hyper-competitive environments. It suggests that “lying flat” might function as a defensive coping mechanism rather than an indicator of complete psychological defeat, a posture conceptually echoing defensive pessimism where individuals strategically lower expectations to manage anxiety and protect self-worth (Norem and Cantor, 1986). It represents a deliberate, strategic detachment—akin to adaptive psychological detachment from chronic stressors (Sonnentag and Fritz, 2015)—where individuals voluntarily lower their external goal pursuits to preserve their internal sense of competence and shield their positive self-esteem from the exhaustive demands of academic “involution.” Consequently, our parallel mediation model reveals that gratitude is associated with mental health through two distinct, non-interfering pathways: a behavioral reservoir associated with lower maladaptive passive withdrawal, and a cognitive reservoir that proactively maintains self-positive regard. This concurrent accumulation of distinct resources perfectly aligns with the “resource caravans” corollary within recent advancements of Conservation of Resources theory (Hobfoll et al., 2018).

### 4.4 Theoretical implications: a “defensive decoupling” framework

Beyond the direct validation of our specific hypotheses, the confirmation of the robust parallel mediation model—and the empirical rejection of a sequential loss spiral—contributes a novel theoretical perspective. Traditionally, behavioral withdrawal postures like Tangping or “quiet quitting” are uniformly pathologized as a maladaptive collapse of the self-system. However, our findings empirically support a “defensive decoupling” mechanism within hyper-competitive educational environments (neijuan). By demonstrating that the Tangping tendency and positive self-esteem operate as independent pathways, we suggest that voluntary psychological detachment can serve as a strategic, rational firewall rather than an indicator of psychological defeat. In this context, gratitude emerges not as a superficial tool to coerce students back into exhaustive and potentially toxic competition, but as a vital psychological anchor. It acts bilaterally: being associated with lower pathological extremes of passive withdrawal while concurrently and independently being associated with higher core self-worth. Consequently, this dual-pathway model demands a pivot in theoretical frameworks—shifting from demanding relentless, unconditional resilience to understanding how individuals create psychological safety to disengage from systemic pressures without compromising their fundamental self-esteem.

### 4.5 Practical implications

The parallel mediation findings observed in this study suggest targeted practical insights for higher education interventions. Rather than viewing the Tangping subculture merely as a behavioral issue to be reprimanded, colleges should adopt a dual-target approach that simultaneously addresses adaptive coping and self-concept preservation. Universities could develop ‘Gratitude and Cognitive-Behavioral Resilience’ structured programs (e.g., comprehensive 6-week interventions), which have been empirically proven to yield sustainable mental health benefits (Bohlmeijer et al., 2021). Encouraging regular gratitude journaling within these programs can help students accumulate positive psychological resources, a pedagogical strategy well-supported by both classroom-based empirical evidence (Flinchbaugh et al., 2012) and robust meta-analytic findings on gratitude well-being associations (e.g., Portocarrero et al., 2020; Dickens, 2017). These resources, as our model indicates, work bilaterally: they are associated with lower maladaptive behavioral withdrawal of Tangping, while concurrently and independently being associated with higher positive self-esteem. Furthermore, from a systemic perspective, these findings imply that educational administrators should consider policies that mitigate excessive academic ‘involution,’ thereby addressing the environmental stressors that trigger the defensive Tangping posture from the root.

### 4.6 Limitations and future directions

Despite its conceptual and empirical contributions, this study has several limitations that warrant future investigation. First, the cross-sectional design precludes the determination of absolute causality. While the hypothesized pathways are grounded in rigorous theoretical frameworks (e.g., Broaden-and-Build theory, Social Cognitive Theory), several alternative model configurations remain plausible. For instance, positive self-esteem could function as an antecedent of gratitude rather than a consequence. However, intervention studies have demonstrated that cultivating gratitude produces gains in psychological well-being (Bohlmeijer et al., 2021; Dickens, 2017), supporting the gratitude → well-being direction specified in our model. Similarly, better mental health could reduce the tendency toward Tangping, rather than the reverse. However, Conservation of Resources theory positions personal resources as temporally preceding coping responses (Hobfoll, 1989), consistent with the gratitude → Tangping pathway. Tangping might also operate as a moderator, such that the gratitude–mental health association is weaker among students with more pronounced Tangping tendencies. However, prior research has predominantly conceptualized Tangping as a mediating mechanism through which psychological resources relate to mental health (Lu et al., 2024), rather than as a boundary condition. Although the present model represents the most theoretically grounded configuration, future research should employ cross-lagged panel designs or randomized controlled trials (RCTs) of gratitude interventions to disentangle these competing directional hypotheses and establish temporal precedence. Second, reliance on self-report data introduces the risk of common method variance (CMV) and social desirability bias. Although our preliminary Harman’s single-factor test and rigorous procedural controls indicated that CMV was not a severe contaminant, the stigma associated with “lying flat” in diligence-oriented cultures may still lead to subtle underreporting. Future studies would benefit from incorporating ecological momentary assessment (EMA) or multi-informant approaches (e.g., peer or instructor ratings) to capture more objective data. Third, the convenience sample was predominantly female (80.4%) and drawn from humanities and social science disciplines (78.5%), which may limit the external validity of the findings. Preliminary analyses revealed that gender was significantly associated with both the Tangping tendency and positive self-esteem, and that academic discipline was significantly associated with positive self-esteem. These demographic differences raise the possibility that the proposed mediation model may not operate identically across subgroups; for example, the protective role of positive self-esteem may be more pronounced among female students or within particular disciplinary contexts. Although the present sample size within male and STEM subgroups was insufficient to support adequately powered multi-group comparisons, future research with more balanced, diverse samples should explicitly test for measurement invariance and structural invariance across gender, discipline, and institutional types to determine the boundary conditions of the model. Finally, the Tangping tendency is a multifaceted construct encompassing distinct sub-dimensions (e.g., cognitively awakened vs. behaviorally escapist Tangping). Utilizing a composite score in this initial mediation model might mask dimension-specific effects on psychological functioning; therefore, a more granular dimensional analysis should be a priority for future psychometric and empirical endeavors.

## Data Availability

The original contributions presented in the study are included in the article/Supplementary material, further inquiries can be directed to the corresponding author/s.
