# Supplementary material for: Dual protective pathways: parallel mediating roles of Tangping tendency and positive self-esteem between gratitude and mental health among Chinese college students
Source: Front Psychol. 2026 Jun 16;17:1853087. doi: 10.3389/fpsyg.2026.1853087 (PMC13314408; doi:10.3389/fpsyg.2026.1853087)
Supplement: Supplementary file 1 [file Table_1.DOCX]

Supplementary Material


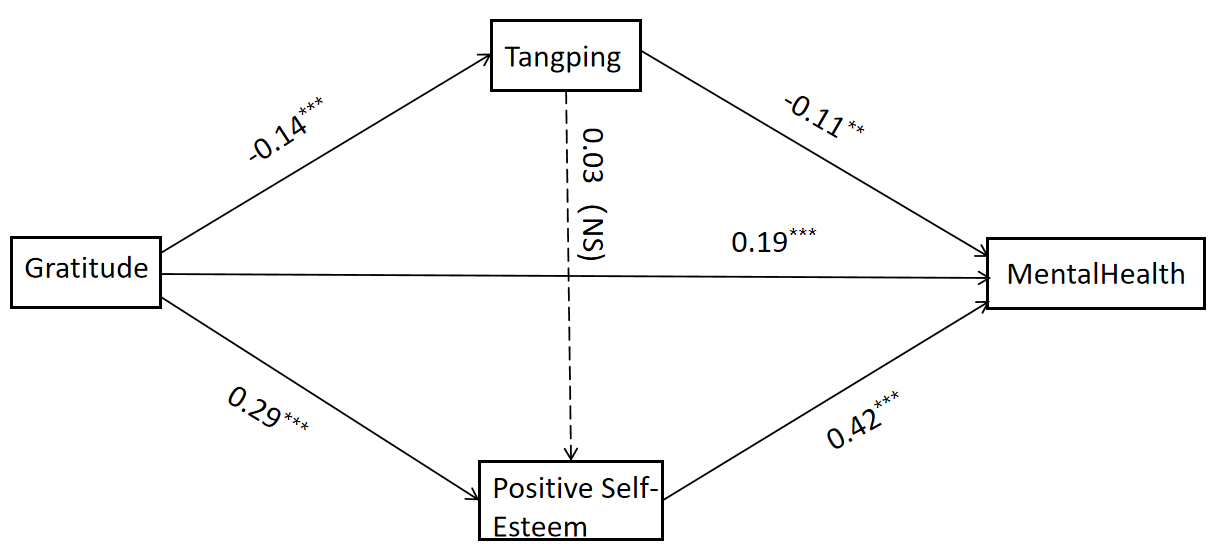
**Supplementary Figure 1**

Figure 1. Parallel mediation model demonstrating the relationships among gratitude, Tangping tendency, positive self-esteem, and mental health.

Values presented are standardized regression coefficients (β). The non-significant path from Tangping tendency to positive self-esteem (β = .03) is indicated by a dashed line. Gender and academic discipline were controlled as covariates.

*** *p* < .001.
